# Impact of Dyspnea on Adults With Respiratory Symptoms Without a Defined Diagnosis

**DOI:** 10.1016/j.chest.2024.07.183

**Published:** 2024-09-04

**Authors:** Jared Bierbrier, Emily Gerstein, George A. Whitmore, Katherine L. Vandemheen, Celine Bergeron, Louis-Philippe Boulet, Andreanne Cote, Stephen K. Field, Erika Penz, R. Andrew McIvor, Catherine Lemière, Samir Gupta, Paul Hernandez, Irvin Mayers, Mohit Bhutani, M. Diane Lougheed, Christopher J. Licskai, Tanweer Azher, Nicole Ezer, Martha Ainslie, Gonzalo G. Alvarez, Sunita Mulpuru, Shawn D. Aaron

**Affiliations:** a The Ottawa Hospital Research Institute, University of Ottawa, Ottawa, ON, Canada; b Desautels Faculty of Management, McGill University, Montreal, QC, Canada; c Department of Medicine, The University of British Columbia, Vancouver, BC, Canada; d Centre de recherche, Institut de cardiologie et de pneumologie de Québec, Université Laval, Quebec, QC, Canada; e Cumming School of Medicine, University of Calgary, Calgary, AB, Canada; f Department of Medicine, University of Saskatchewan, Regina, SK, Canada; g Firestone Institute for Respiratory Health, McMaster University, Hamilton, ON, Canada; h Department of Medicine, Université de Montreal, Montreal, QC, Canada; i Department of Medicine and the Li Ka Shing Knowledge Institute, St. Michael’s Hospital University of Toronto, Toronto, ON, Canada; j Department of Medicine, Dalhousie University, Halifax, NS, Canada; k Department of Medicine, University of Alberta, Edmonton, AB, Canada; l Department of Medicine, Queen’s University, Kingston, ON, Canada; m Department of Medicine, University of Western Ontario, London, ON, Canada; n Department of Medicine, Memorial University, St. John’s, Newfoundland, NL, Canada; o Department of Medicine, McGill University, Montreal, QC, Canada; p Department of Medicine, University of Manitoba, Winnipeg, MB, Canada

**Keywords:** asthma, case finding, COPD, dyspnea

## Abstract

**Background:**

We investigated dyspnea; its associated risk factors; and its impact on health care utilization, quality of life, and work productivity in adults with undiagnosed respiratory symptoms.

**Research Question:**

What is the impact of dyspnea in adults with undiagnosed respiratory symptoms?

**Study Design and Methods:**

This population-based study included 2,857 adults who were experiencing respiratory symptoms. These individuals had not been previously diagnosed with any lung conditions and were recruited from 17 Canadian centers using random digit dialing. Each participant underwent spirometry testing both before and after using a bronchodilator to determine if they met the diagnostic criteria for COPD, asthma, or preserved ratio impaired spirometry (PRISm), or if their spirometry results were normal. An age-matched control group (n = 231) was similarly recruited using random digit dialing. A dyspnea impact assessment score from 0 to 100 was produced using questions from the COPD Assessment Test and St. George’s Respiratory questionnaire.

**Results:**

Individuals with PRISm (n = 172) reported more impactful dyspnea (mean score, 63.0; 95% CI, 59.5-66.4) than those with undiagnosed asthma (n = 265; mean score, 56.6; 95% CI, 53.9-59.3) or undiagnosed COPD (n = 330; mean score, 57.5; 95% CI, 55.1-59.9). All groups reported significantly more impactful dyspnea than the control group (mean score, 13.8; 95% CI, 11.8-15.7). Patient-specific risk factors including age, sex, BMI, smoking, and comorbidities explained 20.6% of the variation in dyspnea. An additional 12.4% of the variation was explained by disease classification and another 1.7% by the severity of lung function impairment assessed with spirometry. After adjusting for age, sex, and BMI, greater dyspnea impact was associated with increased health care utilization, lower quality of life, and reduced work productivity.

**Interpretation:**

Our findings showed that in community-based adults with undiagnosed respiratory symptoms, those identified with PRISm experienced the greatest impact of dyspnea. Dyspnea imposes burdens on the health care system and is associated with impaired quality of life and work productivity.

FOR EDITORIAL COMMENT, SEE PAGE 1259

**Take-home Points**

**Study Question:** How profoundly are adults with undiagnosed respiratory symptoms affected by dyspnea?

**Results:** In community-based adults with undiagnosed respiratory symptoms, those identified with preserved ratio impaired spirometry experienced the greatest impact of dyspnea, followed by those with undiagnosed asthma or COPD. Greater dyspnea impact was associated with increased health care utilization, lower quality of life, and reduced work productivity.

**Interpretation:** Dyspnea imposes burdens on the health care system and is associated with impaired quality of life and work productivity.

Dyspnea refers to a subjective sensation of breathing discomfort.^1^ In a study involving a community-based population aged > 70 years, the prevalence of dyspnea was found to be 32%.^2^ Dyspnea can lead to limitations in daily activities, reduced exercise tolerance, and heightened mortality risks.^3^

Dyspnea not only affects individuals with diagnosed respiratory conditions but also poses a significant burden on those with undiagnosed conditions. In a systematic review by Müller et al,^4^ the combined prevalence of dyspnea in the adult general population across 11 studies was estimated to be 10%. Dyspnea can arise from a broad spectrum of underlying factors, including both respiratory and nonrespiratory conditions. Studies have revealed that dyspnea is not solely attributable to respiratory conditions but is also heavily influenced by cardiovascular deconditioning and by nonrespiratory factors, including psychosocial, social, and environmental determinants.^5^^,^^6^

Dyspnea is a prevalent symptom with consequences that extend beyond its physiologic implications. A study in European patients with COPD explored the burden of dyspnea and identified potential correlates. The study revealed that higher dyspnea impact correlated with lower health-related quality of life, increased work impairment, and a higher frequency of emergency department visits.^7^

The three objectives of our study were as follows: (1) to evaluate the impact of dyspnea in adults from the general population who had no prior diagnosis of respiratory disease but who reported having significant respiratory symptoms in the past 6 months; (2) to identify associated risk factors for dyspnea and estimate their influence on the symptom; and (3) to explore the relationship between dyspnea and health care utilization, quality of life, and work productivity in adults with undiagnosed respiratory symptoms.

## Study Design and Methods

### Recruitment of Undiagnosed Cases and Healthy Control Patients

Between June 2017 and January 2023, adults aged ≥ 18 years were recruited through a two-step process into the Undiagnosed COPD and Asthma Population (UCAP) study, a multicenter case finding study. Approval for the study was obtained from the research ethics boards of the 17 participating study sites across Canada. Informed, written consent was provided by all study participants.

Both landlines and cellphones within a 90-minute radius of any of the 17 study sites were dialed randomly. A prerecorded message then inquired whether any household member was ≥ 18 years of age and had experienced respiratory symptoms (eg, shortness of breath, wheezing, increased mucus or sputum, prolonged cough) within the past 6 months. Households with affirmative responses were subsequently contacted by the local study coordinator for a follow-up call. The household member reporting respiratory symptoms was verbally consented and screened for eligibility to participate in the study over the telephone.^8^^,^^9^

Exclusion criteria included the following: (1) a history of diagnosis of lung or airway disease, (2) use of respiratory inhalers aside from as-needed salbutamol, (3) contraindications for spirometry (eg, occurrences of myocardial infarction, stroke, aortic or cerebral aneurysm, eye surgery, detached retina within the last 3 months), (4) inability or refusal to provide informed consent, (5) being in the third trimester of pregnancy, and (6) being < 18 years of age.

Each participant completed the Asthma Screening Questionnaire (ASQ)^10^ via telephone. Individuals aged ≥ 60 years, and those aged < 60 years who scored < 6 points on the ASQ, also completed the COPD-Diagnostic Questionnaire.^11^^,^^12^ Participants scoring ≥ 6 points on the ASQ or ≥ 20 points on the COPD-Diagnostic Questionnaire were invited to the study site for pre- and post-bronchodilator (BD) spirometry.

A control group without respiratory symptoms was selected randomly using identical random digit dialing methods. Control patients reported no respiratory symptoms in the preceding 6 months and obtained a score of 0 on the ASQ. Participants were recruited as control patients if they could be matched with an individual from the undiagnosed group based on age (± 5 years) and sex. This matching process aimed to have similar demographic profiles between the control group and the newly found cases. This matching was implemented solely to ensure demographic comparability across the study groups and not for pairing patients for statistical analysis purposes.

All participants filled out the COPD Assessment Test (CAT) questionnaire. Elevated CAT scores indicate a greater burden of respiratory symptoms impacting daily activities and health status.^13^ The St. George’s Respiratory Questionnaire (SGRQ)^14^, ^15^, ^16^ was used to assess respiratory disease-related quality of life. Higher SGRQ scores indicate poorer health status. Both the CAT and SGRQ questionnaires were completed prior to spirometry to avoid influencing patients’ perceptions of their dyspnea.

### Classification of Undiagnosed Cases

Certified study personnel administered spirometry tests before and after BD use. Participants showing an increase of at least 12% and 200 mL in their FEV_1_ after receiving 400 μg of salbutamol were classified as having spirometry indicative of asthma.^17^ Those whose post-BD ratio of FEV_1_/FVC fell below the lower 95% confidence limit (ie, FEV_1_/FVC < lower limit of normal) were classified as having spirometry indicative of COPD.^18^ Participants meeting the criteria for both conditions were labeled as having COPD. Those with a post-BD FEV_1_ < 80% of the predicted normal and a post-BD FEV_1_/FVC ratio > 0.70 were classified as having spirometry indicative of preserved ratio impaired spirometry (PRISm). PRISm was defined based on post-BD spirometry values for a more specific classification.^19^ Participants not meeting criteria for asthma, COPD, or PRISm were labeled as having normal spirometry.

### Assessment of the Impact of Participants’ Dyspnea

Although neither the CAT nor the SGRQ are dyspnea-specific tools, both are recommended by the Global Initiative for Chronic Obstructive Lung Disease to evaluate symptoms, including dyspnea,^20^ and both yield a richer assessment of dyspnea than the modified Medical Research Council breathlessness scale.^20^ Fifteen questions were taken from the CAT and SGRQ questionnaires that referred to individuals’ experiences with dyspnea, and a composite measure of dyspnea impact using a weighted sum of the responses to the 15 questions was constructed. Questions were coded so that larger values indicate more impactful dyspnea. Weights used for question responses in calculating the dyspnea impact assessment measure were those of the first component of a principal component analysis (PCA) based on the covariance matrix of question responses. Questions with multiple responses and ordinal structure are individually more informative and thus were accorded higher weight than individual true-false questions. No additional PCA component was anticipated a priori to be material for our investigation, and an eigenvalue analysis of the PCA was conducted to verify this assumption.

The composite dyspnea impact measure was scaled so its minimum value was 0 if the response to each of the 15 questions was 0, and the maximum value was scaled to 100 if the individual responses for all 15 questions represented the most severe dyspnea response.

### Risk Factors Associated With Dyspnea

Patient-related risk factors were considered first, and results of spirometry considered afterward. The spirometry risk factors chosen for the second stage analysis included the spirometry-based diagnosis of the patient (asthma, COPD, PRISm, or normal) and lung function results indicative of the severity of physiologic impairment. Severity was gauged by assessing three principal lung function measures: (1) post-BD FEV_1_ % predicted, (2) post-BD FEV_1_/FVC ratio, and (3) percentage reversal of FEV_1_ with BD.

### Dyspnea Impact and Health Care Use, Quality of Life, and Work Productivity

The impact of dyspnea and its associations with health care use, quality of life, and work productivity were examined. Health care utilization was assessed through self-reported data. Quality of life was assessed using the 36-Item Short Form Health Survey questionnaire, where higher scores indicate better health status. Work productivity was assessed using the Work Productivity and Activity Impairment questionnaire, where higher scores indicate greater impairment in work productivity and daily activities.

### Statistical Analysis

Box plots were used to compare distribution patterns of dyspnea impact assessments among the disease groups. Pairwise comparison tests were conducted to evaluate mean dyspnea differences between groups. Multiple linear regression analysis was used to measure contributions to variability of dyspnea by selected patient-specific risk factors, spirometry disease classification, and key lung function measures. The selected sets of risk factors were evaluated using successive regression analyses. Analysis of variance sums of squares from the successive regression analyses provided the cumulative percentage contributions to variability of dyspnea. Simple, multiple, and logistic regression analyses were used to study associations between dyspnea and health care utilization, quality of life, and work productivity outcomes. All statistical analyses were done using STATA 16 statistical software (StataCorp).

## Results

**Figure 1:**
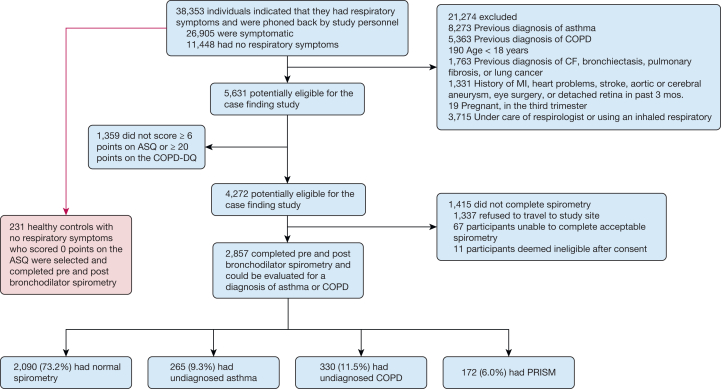
Study flow diagram demonstrating the case finding and control group recruitment and allocation. ASQ = Asthma Screening Questionnaire; COPD-DQ= COPD Diagnostic Questionnaire; CF = cystic fibrosis; MI = myocardial infarction; PRISM = preserved ratio impaired spirometry.

Figure 1 illustrates the results of the case finding approach, including the enrollment of the control group. Among 5,631 potentially eligible participants, 1,359 participants (24%) did not meet the threshold of ≥ 6 points on the ASQ or ≥ 20 points on the COPD-Diagnostic Questionnaire and were thus excluded, leaving 4,272 individuals deemed eligible for spirometry. However, 1,415 either did not attend or were unable to complete adequate spirometry. Ultimately, 2,857 (67%) of those eligible underwent both pre- and post-BD spirometry.

Of these 2,857 participants, 2,090 (73.2%) had normal spirometry, 265 (9.3%) had undiagnosed asthma, 330 (11.5%) had undiagnosed COPD, and 172 (6.0%) had PRISm based on post-BD spirometry. Of the 595 individuals with spirometric evidence of asthma or COPD, 253 were independently assessed by a pulmonologist. In 245 of these 253 cases (97%), the independent physician diagnosis agreed with the study diagnosis of asthma or COPD.

Individuals in the COPD group were generally older and more likely to be male compared with all other study groups (Table 1). All groups, including healthy control participants, had mean BMIs in the overweight or obese ranges. The PRISm group was heaviest with an average BMI of 34.7, and 22% of PRISm patients met BMI criteria for morbid obesity. Compared with all other groups, those with COPD were the most likely to have active or previous tobacco use, with the highest average total pack-years of 32.7. The control group had the lowest number of people with active or previous tobacco use.

**Table 1:** Descriptive Characteristics and Demographics of the Study Group

Descriptive Characteristic	Control Group (n = 231)	Normal Spirometry Group (n = 2,090)	Asthma Group (n = 265)	COPD Group (n = 330)	PRISm Group (n = 172)
Age, y	61.5 (14.6)	59.2 (15.3)	58.7 (16.0)	66.1 (11.3)	60.9 (14.4)
Female, No. (%)	98 (42)	1082 (52)	116 (44)	121 (37)	86 (50)
Income, No. (%)					
< CAD $30,000	10 (4.5)	209 (10)	18 (7)	51 (15)	34 (20)
≥ CAD $30,000	211 (91)	1522 (73)	204 (77)	213 (65)	106 (62)
Income not reported	10 (4.5)	359 (17)	43 (16)	66 (20)	32 (18)
BMI, kg/m^2^	28.35 (5.39)	30.50 (6.60)	30.65 (7.03)	28.57 (5.86)	34.66 (8.80)
BMI, No. (%)					
Not overweight	68 (29)	422 (20)	60 (23)	98 (30)	20 (12)
Overweight	86 (37)	675 (32)	72 (27)	127 (38)	37 (21)
Obese	71 (31)	812 (39)	104 (39)	91 (28)	77 (45)
Morbidly obese	6 (3)	181 (9)	29 (11)	14 (4)	38 (22)
Prebronchodilator spirometry					
FEV_1_, L	2.98 (0.76)	2.89 (0.81)	2.53 (0.78)	2.01 (0.71)	2.06 (0.56)
FEV_1_, % predicted	102.5 (15.0)	99.6 (14.0)	83.7 (13.6)	70.3 (17.0)	70.7 (7.7)
FEV_1_/FVC	0.74 (0.07)	0.76 (0.06)	0.69 (0.07)	0.58 (0.08)	0.77 (0.06)
Post-bronchodilator spirometry					
FEV_1_, L	3.01 (0.8)	2.98 (0.84)	2.89 (0.86)	2.16 (0.71)	2.09 (0.57)
FEV_1_ % predicted	105.9 (14.6)	102.6 (14.0)	95.6 (14.4)	75.5 (16.0)	71.6 (7.3)
FEV_1_/FVC	0.77 (0.07)	0.79 (0.06)	0.74 (0.06)	0.60 (0.09)	0.78 (0.06)
Change in FEV_1_ postbronchodilator, %	3.4 (4.7)	3.0 (3.7)	11.9 (3.9)	5.0 (5.4)	0.9 (3.9)
Comorbidities, No. (%)					
Congestive heart failure	1 (0.4)	22 (1)	5 (2)	10 (3)	9 (5)
Coronary artery disease	24 (10)	180 (9)	24 (9)	64 (19)	31 (18)
Depression/anxiety	70 (30)	836 (40)	106 (40)	99 (30)	54 (31)
Diabetes mellitus	23 (10)	261 (12)	33 (12)	42 (13)	45 (26)
Hypertension	66 (29)	704 (34)	94 (35)	122 (37)	86 (50)
Anemia	31 (13)	351 (17)	44 (17)	42 (13)	23 (13)
Cancer	23 (10)	222 (11)	25 (9)	51 (15)	13 (8)
GERD	55 (24)	735 (35)	89 (34)	98 (30)	63 (37)
Liver disease	6 (3)	89 (4)	12 (5)	14 (4)	9 (5)
Renal disease	10 (4)	85 (4)	9 (3)	20 (6)	10 (6)
Stroke	5 (2)	72 (3)	5 (2)	15 (5)	11 (6)
Smoking status, No. (%)					
Does not smoke	149 (65)	912 (44)	104 (39)	44 (13)	72 (42)
Previous tobacco use	77 (33)	839 (40)	118 (45)	158 (48)	68 (40)
Active tobacco use	4 (2)	324 (16)	41 (16)	128 (39)	29 (17)
Total pack-y	5.6 (11.8)	10.7 (16.5)	12.7 (17.9)	32.7 (27.2)	13.3 (19.6)
Total months of occupational exposure^a^	36.5 (112.5)	74.7 (193.1)	101.1 (278.9)	119.5 (262.4)	99.3 (263.2)

Data are presented as mean (SD) unless otherwise stated. GERD = gastroesophageal reflux disease; PRISm = preserved ratio impaired spirometry.

a Occupational exposure includes hard-rock mining, coal mining, sandblasting, working with asbestos, chemical/plastics manufacturing, flour/feed/grain milling, cotton/jute processing, foundry/steel milling, welding, fire fighting, farming, forestry, saw-milling, and working with paint/chemicals/fumes.

Table 2 shows mean responses to the 15 dyspnea questions for each disease classification and presents question weights (PCA scoring coefficients) used for calculating the dyspnea impact assessment.

**Table 2:** Mean Responses to Individual Dyspnea Questions

Questions About Dyspnea From CAT and SGRQ		Control Group (n = 231)	Normal Spirometry Group (n = 2,090)	Asthma Group (n = 265)	COPD Group (n = 330)	PRISm Group (n = 172)
Q1 (weight = 0.514)	When I walk up a hill or one flight of stairs, I am breathless… The scale for this question ranges from 0 (when I walk up a hill or 1 flight of stairs, I am not breathless) to 5 (when I walk up a hill or one flight of stairs, I am very breathless).	0.90 (1.04)	2.85 (1.46)	3.03 (1.37)	3.21 (1.30)	3.56 (1.37)
Q2 (weight = 0.436)	Over the past 3 mo, I have had shortness of breath… The scale for this question ranges from 0 (over the past 3 mo, I have had shortness of breath…not at all) to 4 (over the past 3 mo, I have had shortness of breath…most days a week).	0.45 (0.89)	2.50 (1.30)	2.71 (1.18)	2.83 (1.21)	2.93 (1.18)
Q3: I feel breathless these days…						
	Sitting or lying still, %	3	16	23	14	19
	Getting washed or dressed, %	2	17	21	20	28
	Walking around at home, %	2	20	21	23	27
	Walking outside on the level, %	4	36	42	38	49
	Climbing up a flight of stairs, %	20	75	81	83	87
	Climbing hills, %	35	83	89	90	89
	Playing sports or games, %	34	78	83	81	82
Q3 (total) (weight = 0.648)	The scale for this question ranges from 0 to 7, based on the number of positive answers for the 7 items.	1.00 (1.25)	3.23 (1.72)	3.55 (1.63)	3.45 (1.61)	3.76 (1.75)
Q4 (weight = 0.091)	I am breathless when I talk, %	2	35	43	37	39
Q5 (weight = 0.095)	I am breathless when I bend over, %	5	37	45	37	56
Q6 (weight = 0.060)	I get afraid or panic when I cannot get my breath, %	4	30	33	31	37
Because of my breathing…						
Q7 (weight = 0.037)	I take a long time to get washed or dressed, %	1	8	9	10	17
Q8 (weight = 0.023)	I cannot take a bath or shower, or I take a long time, %	0	5	7	7	8
Q9 (weight = 0.116)	I walk slower than other people, or I have to stop for rests, %	5	40	46	56	66
Q10 (weight = 0.113)	Jobs such as housework take a long time, or I have to stop for rests, %	3	38	40	48	59
Q11 (weight = 0.124)	If I climb up one flight of stairs, I have to go slowly or stop, %	5	47	44	57	67
Q12 (weight = 0.127)	If I hurry or walk fast, I have to stop or slow down	10	59	62	70	80
Q13 (weight = 0.132)	My breathing makes it difficult to do things such as climbing up hills, carrying things up stairs, light gardening such as weeding, dancing, bowling, or golfing, %	8	54	59	69	74
Q14 (weight = 0.123)	My breathing makes it difficult to do things such as carrying heavy loads, digging the garden or shoveling snow, jogging, or walking at 5 km/h, playing tennis or swimming, %	13	65	71	78	81
Q15 (weight = 0.108)	My breathing makes it difficult to do things such as very heavy manual work, running, cycling, swimming fast, or playing competitive sports, %	17	74	79	85	88

Data are presented as mean (SD) for Q1, Q2, and Q3 (total), and Q3 to Q15 were presented to participants as yes or no questions, where percentages of participants who answered yes are shown. Question weights (principal component analysis scoring coefficients) used for calculating the dyspnea assessment are shown below individual questions. CAT = COPD Assessment Test; PRISm = preserved ratio impaired spirometry; Q = question; SGRQ = St. George’s Respiratory Questionnaire.

**Figure 2:**
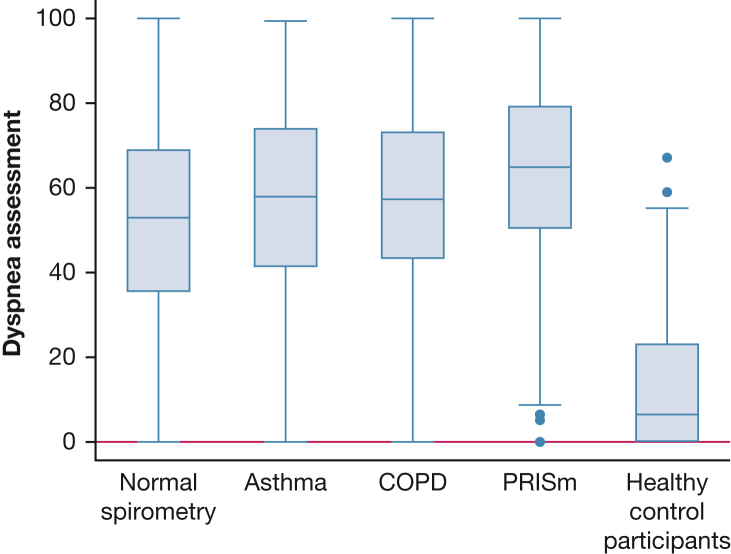
Box plot demonstrating dyspnea impact according to spirometry disease classification. The center line marks the median. The boxes span the interquartile range (IQR). The outer fences are set at distances 1.5 × IQR from the box. Outliers appear as plotted dots.

Individuals with PRISm reported the highest dyspnea impact, with a significantly greater mean score (63.0; 95% CI, 59.5-66.4) than those with undiagnosed asthma or COPD (Table 3). Those with undiagnosed asthma or COPD had similar mean scores (56.6; 95% CI, 53.9-59.3 and 57.5; 95% CI, 55.1-59.9, respectively), followed by those with normal spirometry (51.8; 95% CI, 50.7-52.8). All four groups reported significantly more impactful dyspnea than the control group (mean score, 13.8; 95% CI, 11.8-15.7). Table 3 shows between-group differences in mean dyspnea impact assessments for each pair of disease outcomes. Figure 2 compares box plots of the dyspnea impact assessment values across disease classifications.

**Table 3:** Intergroup Comparisons of Dyspnea Impact

Pairwise Comparison	Mean Dyspnea Score (95% CI)	Mean Difference (95% CI)	*P* Value
Control	13.8 (11.8-15.7)	−38.0 (−41.1 to −34.9)	< .001
Normal spirometry	51.8 (50.7-52.8)		
Control	13.8 (11.8-15.7)	−43.7 (−47.6 to −39.8)	< .001
COPD	57.5 (55.1-59.9)		
Control	13.8 (11.8-15.7)	−42.8 (−46.9 to −38.7)	< .001
Asthma	56.6 (53.9-59.3)		
Control	13.8 (11.8-15.7)	−49.2 (−53.7 to −44.6)	< .001
PRISm	63.0 (59.5-66.4)		
Normal spirometry	51.8 (50.7-52.8)	5.7 (3.0 to 8.4)	< .001
COPD	57.5 (55.1-59.9)		
Normal spirometry	51.8 (50.7-52.8)	4.8 (1.8, 7.8)	.002
Asthma	56.6 (53.9-59.3)		
Normal spirometry	51.8 (50.7-52.8)	11.2 (7.5 to 14.8)	< .001
PRISm	63.0 (59.5-66.4)		
PRISm	63.0 (59.5-66.4)	5.5 (1.1 to 9.8)	.014
COPD	57.5 (55.1-59.9)		
PRISm	63.0 (59.5-66.4)	6.4 (1.9 to 10.9)	.005
Asthma	56.6 (53.9-59.3)		
Asthma	56.6 (53.9-59.3)	0.9 (−2.8 to 4.7)	.63
COPD	57.5 (55.1-59.9)		

PRISm = preserved ratio impaired spirometry.

Table 4 presents the association of dyspnea with patient-specific risk factors. Dyspnea impact increased with younger age, being female, higher BMI, higher smoking and smoke exposure history, and total work exposure in an array of risky occupations. These risk factors, taken as a whole, accounted for 21% of the variability in dyspnea.

**Table 4:** Sequential Regression Analyses of Risk Factors Contributing to Variability in Dyspnea: Dyspnea Regressed on Patient-Specific Risk Factors (20.6% of Variability Explained)

Risk Factor	Regression Coefficient	*P* Value
Age	−0.0909	**.005**
Female	8.217	**< .001**
BMI	0.899	**< .001**
Household income < CAD $30,000	1.420	.40
Household income ≥ CAD $30,000	−2.149	.07
Smoking history, pack-y	0.144	**< .001**
Smoking exposure	5.123	**< .001**
Occupational exposure	0.00975	**< .001**
Congestive heart failure	10.119	**.004**
Coronary artery disease	4.813	**.001**
Depression/anxiety	6.892	**< .001**
Diabetes mellitus	1.627	.22
Hypertension	3.433	**< .001**
Anemia	1.738	.15
Cancer	0.952	.49
GERD	4.663	**< .001**
Liver disease	1.081	.61
Renal disease	2.073	.32
Stroke	8.463	**< .001**

Boldface indicates statitistical significance. GERD= gastroesophageal reflux disease.

After adjustment for patient-specific risk factors in the first stage analysis, we adjusted for spirometry-defined disease (PRISm, asthma, COPD, or normal spirometry) in Table 5. Adjustment for disease classification accounted for 12% of the total variability of dyspnea.

**Table 5:** Dyspnea Regressed on Spirometry Disease Group

Disease Group	Regression Coefficient	*P* Value
Control	−31.2	**< .001**
Normal spirometry^a^	NA	NA
Asthma	4.6	**.001**
COPD	3.8	**.003**
PRISm	5.5	**.001**
Constant	51.9	NA

Dyspnea regressed on spirometry disease group, after removing contributions from subject-specific factors in Table 4 (12.4% of variability explained). Boldface indicates statitistical significance. NA = not applicable; PRISm = preserved ratio impaired spirometry.

a Normal spirometry group is the reference category.

Table 6 presents the contribution of lung function measures of physiologic impairment after accounting for patient-related risk factors and disease classification. For the PRISm disease group, a higher post-BD FEV_1_/FVC ratio and a lower post-BD FEV_1_ % predicted value were associated with greater dyspnea impact. For the COPD disease group, a lower post-BD FEV_1_/FVC ratio was associated with greater dyspnea impact. Reversibility of FEV_1_ was associated with higher dyspnea impact only in patients with asthma or COPD. Lung function measures of disease severity accounted for 2% of the variability in dyspnea.

**Table 6:** Dyspnea Regressed on Lung Function Variables Representing Severity of Impairment

Disease Group	Reversibility of FEV_1_, %	Post-BD FEV_1_/FVC Ratio	Post-BD FEV_1_ % predicted	Overall *P* Value
Control	−0.163 (*P* = .47)	−0.274 **(*P* = .05)**	−0.090 (*P* = .17)	.096
Normal spirometry	0.186 (*P* = .16)	0.240 **(*P* = .005)**	−0.131 **(*P* < .001)**	**< .001**
Asthma	0.545 **(*P* = .01)**	0.107 (*P* = .58)	−0.158 (*P* = .08)	**.009**
COPD	0.392 **(*P* = .002)**	−0.307 **(*P* = .05)**	−0.075 (*P* = .37)	**< .001**
PRISm	−0.290 (*P* = .39)	0.854 **(*P* = .002)**	−0.650 **(*P* = .004)**	**< .001**

Dyspnea regressed on lung function variables representing severity of impairment, after removing contributions of patient-specific factors and spirometry disease group Tables 4 and 5 (1.7% of variability explained). Boldface indicates statitistical significance. BD = bronchodilator; PRISm = preserved ratio impaired spirometry.

After adjusting for age, sex, and BMI, dyspnea was negatively associated with all domains of quality of life, including physical functioning (coefficient, −0.655; *P* < .001), role limitations due to physical health (coefficient, −0.628; *P* < .001), general health (coefficient, −0.382; *P* < .001), and total score (coefficient, −0.473; *P* < .001) (Table 7).

**Table 7:** Unadjusted and Adjusted Dyspnea Associations With Quality of Life (SF-36)

Measure	Unadjusted		Adjusted	
	Dyspnea Coefficient (95% CI)	*P* Value	Dyspnea Coefficient (95% CI)	*P* Value
Physical functioning	−0.693 (−0.718 to −0.668)	< .001	−0.655 (−0.680 to −0.630)	< .001
Physical health limitations	−0.634 (−0.666 to −0.603)	< .001	−0.628 (−0.661 to −0.595)	< .001
Emotional problems	−0.403 (−0.438 to −0.369)	< .001	−0.407 (−0.443 to −0.370)	< .001
Energy/fatigue	−0.454 (−0.479 to −0.428)	< .001	−0.452 (−0.479 to −0.425)	< .001
Emotional well-being	−0.230 (−0.256 to −0.204)	< .001	−0.239 (−0.266 to −0.213)	< .001
Social functioning	−0.433 (−0.466 to −0.399)	< .001	−0.434 (−0.469 to −0.399)	< .001
Pain	−0.410 (−0.444 to −0.377)	< .001	−0.387 (−0.423 to −0.352)	< .001
General health	−0.390 (−0.416 to −0.364)	< .001	−0.382 (−0.409 to −0.355)	< .001
Total score	−0.485 (−0.504 to −0.467)	< .001	−0.473 (−0.493 to −0.454)	< .001

Adjusted coefficients are adjusted for age, sex, and BMI. Regression coefficients are presented with 95% CIs and *P* values.

After adjusting for age, sex, and BMI, dyspnea was associated with an increased likelihood of annual visits to health care providers for respiratory complaints (OR, 1.011; *P* < .001 for general practitioner visits; OR, 1.015; *P* < .001 for emergency department visits; and OR, 1.023, *P* = .005 for hospitalization for respiratory illness) (Table 8).

**Table 8:** Unadjusted and Adjusted Dyspnea Associations With Health Care Use

Measure	Unadjusted		Adjusted	
	Dyspnea OR (95% CI)	*P* Value	Dyspnea OR (95% CI)	*P* Value
In the past 12 mo, did you visit your general practitioner or a nurse practitioner or another physician at a walk-in clinic for any breathing problems?	1.011 (1.007-1.014)	< .001	1.011 (1.007-1.014)	< .001
In the past 12 mo, did you visit an emergency department for any breathing problems?	1.015 (1.009-1.021)	< .001	1.015 (1.009-1.022)	< .001
In the past 12 mo, were you hospitalized for any breathing problems or respiratory illness?	1.021 (1.006-1.037)	.006	1.023 (1.007-1.039)	.005

Data are presented as OR (95% CI) with *P* values. Adjusted values are adjusted for age, sex, and BMI.

After adjusting for age, sex, and BMI, dyspnea was associated with a reduced likelihood of current employment (OR, 0.993; *P* < .001), increased absenteeism (coefficient, 0.066; *P* < .001), increased presenteeism (coefficient, 0.349; *P* < .001), higher work productivity loss (coefficient, 0.383; *P* < .001), and greater activity impairment (coefficient, 0.501; *P* < .001), as measured by the Work Productivity and Activity Impairment questionnaire^21^ (Table 9).

**Table 9:** Unadjusted and Adjusted Dyspnea Associations With Work Productivity (WPAI)

Measure	Unadjusted		Adjusted	
	Dyspnea OR (95% CI)	*P* Value	Dyspnea OR (95% CI)	*P* Value
Are you currently employed (working for pay)?	0.995 (0.992-0.998)	.002	0.993 (0.990-0.997)	< .001

Measure^a^	Dyspnea Coefficient (95% CI)	*P* Value	Dyspnea Coefficient (95% CI)	*P* Value
Absenteeism	0.061 (0.040-0.083)	<.001	0.066 (0.044-0.089)	< .001
Presenteeism	0.334 (0.293-0.375)	<.001	0.349 (0.306-0.392)	< .001
Work productivity loss	0.368 (0.323-0.413)	<.001	0.383 (0.336-0.430)	< .001
Activity impairment	0.503 (0.463-0.544)	<.001	0.501 (0.458-0.544)	< .001

ORs and regression coefficients are presented with 95% CIs and *P* values. Adjusted coefficients are adjusted for age, sex, and BMI. WPAI = Work Productivity and Activity Impairment questionnaire.

a Measures calculated from WPAI questions.^21^

## Discussion

Our study explored dyspnea in community-based adults with undiagnosed respiratory symptoms identified via case finding. Surprisingly, we found that the dyspnea experienced by those with PRISm had a greater impact on their activities and health status than those with newly diagnosed COPD or asthma.

The prevalence of individuals who were obese and morbidly obese in the PRISm group partially explains the between-group difference in dyspnea. The excess dyspnea seen in the PRISm group when compared with the normal spirometry group is partly explained by patient-specific risk factors, including BMI, which shrink the mean dyspnea differential between the groups from 11.2 to 5.5 points (Table 3, Table 4, Table 5, Table 6). The remaining 5.5-point difference indicates that PRISm patients have excess dyspnea relative to symptomatic individuals with normal spirometry for additional reasons other than obesity.

Approximately 65% of the variability in dyspnea remained unexplained by the factors examined in our study. Most individuals in our study showed normal spirometry results but still carried a substantial burden of dyspnea, an inconsistency that needs explanation. Several factors not included in our analysis may have contributed to the unexplained variation. Environmental factors (eg, air pollution, allergen exposure, seasonal variations in symptoms) are potential contributors to this unexplained variability.^22^ Genetic predispositions could also play a significant role, as suggested by a study that revealed that parents with dyspnea were 1.8 times more likely to have offspring with dyspnea.^23^ Additionally, fitness could be a contributing factor, especially in individuals with undiagnosed PRISm, asthma, or COPD who may restrict their activities to avoid dyspnea, and hence become deconditioned.^6^

There were significant but modest differences in mean dyspnea levels across the 17 study sites (data not shown), which are not explained by the risk factors we accounted for in our study. This finding is not surprising because some of the potential contributing factors previously mentioned and other site-specific factors (eg, climate, air quality/industrialization, socioeconomic status) of the catchment population tend to vary across study sites.

Dyspnea is a complex, subjective symptom that is modified by nonrespiratory factors including psychosocial, social, and environmental influences.^5^ Interindividual variability in the perception of dyspnea, influenced by these nonrespiratory factors, may play an important role. A study conducted by Ziegler et al^24^ assessed the perception of dyspnea in 42 healthy individuals using a standardized inspiratory resistive loading stimulus. The study used the modified Borg scale to measure dyspnea perception levels. Among the participants subjected to the same inspiratory resistive load, 31%, 45%, and 24% of participants classified their level of dyspnea as low, intermediate, and high, respectively. The study revealed that differences between individuals contribute considerable variability to the perception of dyspnea, even among healthy participants.

The affective dimension of dyspnea can be captured using additional questionnaires (eg, Multidimensional Dyspnea Profile, Dyspnea-12). Studies have explored the use of the Multidimensional Dyspnea Profile in outpatients with cardiorespiratory disease^25^ and the Dyspnea-12 in patients with asthma^26^ and found that the affective aspect of dyspnea can significantly influence the impact of dyspnea on health status, irrespective of the intensity of breathlessness.

In those with PRISm, there was a strong, positive association between higher values for the FEV_1_/FVC ratio and dyspnea. For the PRISm group, a higher FEV_1_/FVC ratio may reflect diminished lung compliance due to interstitial lung disease and/or respiratory system restriction due to obesity, which could contribute to worse dyspnea. Conversely, the association of dyspnea with the FEV_1_/FVC ratio was in the opposite direction for those with asthma or COPD, and a lower FEV_1_/FVC ratio correlated with worse dyspnea, as expected.

Our study complements the literature by focusing on adults with undiagnosed respiratory symptoms who were randomly selected and recruited through active case finding in the community. This increases the generalizability of our results to a broader population. Our dyspnea questions were derived from widely used and validated respiratory health questionnaires, and our dyspnea assessment measure is a weighted average of responses to these validated questions. Consequently, the measure has an immediate interpretation in terms of the lived day-to-day experience of individuals.

Our study has limitations. We did not undertake reliability/reproducibility testing of our questionnaire. The dyspnea impact assessment score was statistically associated with increased health care utilization, lower quality of life, and reduced work productivity; therefore, by virtue of this analysis, our questionnaire has construct validity. However, further attempts at external validation of the questionnaire using an independent data set would be important. Health care utilization during the preceding 12 months was assessed on entry into the study, and there is potential for impaired recall of events. Our study may have missed asthma in some participants because bronchial challenge testing was not conducted on those who tested negative for airflow obstruction or BD responsiveness. A previous study showed that an additional diagnostic step incorporating bronchial challenge testing into a case finding strategy identified asthma in 26% of symptomatic individuals who had normal spirometry and no response to BD.^27^

Individuals with undiagnosed respiratory symptoms, determined to have asthma or COPD through spirometry, experience poor health status.^28^ Therefore, the implementation of known treatment approaches for asthma or COPD is important to improve their conditions.^29^ In contrast, those with normal spirometry or PRISm face unclear treatment approaches. Long-acting BD therapy in symptomatic individuals with tobacco exposure with normal spirometry is not effective.^30^ Weight management programs may be useful for individuals who are obese with PRISm-related dyspnea; however, this awaits definitive clinical trials.^31^

Dyspnea was severe and prevalent within our study group; however, it remained undiagnosed. A study conducted by Stefan et al^32^ revealed that physicians underestimated their patients’ dyspnea 37.9% of the time, whereas nurses underestimated it 3.5% of the time. Moreover, many patients limit their physical activities, which lead them to downplay the extent of their dyspnea.^19^ Patient underreporting of symptoms, coupled with inadequate physician-led investigations of symptoms, may explain why dyspnea often goes undiagnosed in the population.^33^

In conclusion, our study measured dyspnea impact in individuals with no preexisting diagnosis of lung disease who reported respiratory symptoms as part of a purposeful case finding strategy. Individuals with PRISm exhibited the greatest impact of dyspnea, even higher than those newly diagnosed with asthma or COPD. After adjusting for patient factors, comorbidities, pulmonary diseases, and severity of lung physiologic impairment, most of the variability in dyspnea remained unexplained. We also showed that dyspnea was associated with increased health care utilization, impaired quality of life, and work productivity.

## Funding/Support

This study is supported by the Canadian Institutes of Health Research [FDN Grant 154322].

## Financial/Nonfinancial Disclosures

None declared.
